# Research on AGV Path Planning Based on Improved DQN Algorithm

**DOI:** 10.3390/s25154685

**Published:** 2025-07-29

**Authors:** Qian Xiao, Tengteng Pan, Kexin Wang, Shuoming Cui

**Affiliations:** School of Intelligent Science Information Engineering, Shenyang University, Shenyang 110044, China; p17515162261@163.com (T.P.); sincowang7@163.com (K.W.); csm990515@163.com (S.C.)

**Keywords:** automatic guided vehicle, path planning, deep Q network, deep reinforcement learning

## Abstract

Traditional deep reinforcement learning methods suffer from slow convergence speeds and poor adaptability in complex environments and are prone to falling into local optima in AGV system applications. To address these issues, in this paper, an adaptive path planning algorithm with an improved Deep Q Network algorithm called the B-PER DQN algorithm is proposed. Firstly, a dynamic temperature adjustment mechanism is constructed, and the temperature parameters in the Boltzmann strategy are adaptively adjusted by analyzing the change trend of the recent reward window. Next, the Priority experience replay mechanism is introduced to improve the training efficiency and task diversity through experience grading sampling and random obstacle configuration. Then, a refined multi-objective reward function is designed, combined with direction guidance, step punishment, and end point reward, to effectively guide the agent in learning an efficient path. Our experimental results show that, compared with other algorithms, the improved algorithm proposed in this paper achieves a higher success rate and faster convergence in the same environment and represents an efficient and adaptive solution for reinforcement learning for path planning in complex environments.

## 1. Introduction

Driven by cutting-edge concepts, such as Industry 4.0, smart logistics, and smart factories, the industrial mobile robot market has seen rapid growth [1]. Automated guided vehicles (AGVs), as a key piece of equipment in this field, are equipped with advanced automatic guided vehicle systems, which can be electromagnetic or optical. They can drive autonomously along a preset path and include a complete safety protection mechanism and a variety of cargo handling functions [2,3,4,5]. The emergence of AGVs has not only effectively reduced the need for intense manual labor in the processes of transferring, loading and unloading, and handling goods but has also significantly improved work efficiency. AGVs can greatly improve the safety of work, especially in dangerous and complex working environments [6,7,8]. AGVs’ path planning and obstacle avoidance algorithms represent core technologies for achieving autonomous navigation, which directly affects whether AGVs can safely and efficiently complete various tasks in complex and changeable environments.

In recent years, with the increasing development of industrial automation and intelligence, path planning and obstacle avoidance algorithms for AGVs have become a research hot spot. The early Dijkstra algorithm systematically solved the single source shortest path problem for the first time and has been widely used in map navigation and robot path planning [9,10]. However, the nondirectional “circular diffusion” search method in large-scale maps suffers from serious computational overlap issues, making it difficult to meet real-time requirements. To overcome the inefficiency of Dijkstra’s algorithm, the A* algorithm proposed in 1968 uses heuristic functions to guide the search direction. By evaluating the priority of each node, it prioritizes searching for nodes that are more likely to approach the target, avoiding blind search and greatly improving search efficiency [11,12,13,14].

Although the A* algorithm performs better in static environments, the design of heuristic functions highly relies on prior knowledge and still faces computational bottlenecks in dynamic or high-dimensional spaces. In this context, random sampling methods represented by Probabilistic Roadmap Method (PRM) have emerged to address path planning problems in high-dimensional space by constructing probabilistic roadmaps. It can avoid an exhaustive search of the entire high-dimensional space, greatly reducing computational complexity. However, it lacks goal orientation, and it is difficult to efficiently approach the optimal path [15]. In recent years, heuristic optimization algorithms with distinct biological characteristics, such as Grey Wolf Optimizer (GWO) and Whale Optimization Algorithm (WOA), have been introduced into the field of path planning; they have strong global search capabilities and simple implementation advantages. These algorithms effectively compensate for the shortcomings of random sampling, but they also suffer from slow convergence and susceptibility to getting stuck in local optima [16,17,18].

With the continuous success of Deep Reinforcement Learning (DRL) algorithms, scholars have begun to attempt to use deep learning models to learn the modes of path planning. Deep learning methods are mainly used for environmental perception by extracting obstacle features in high-dimensional space, and reinforcement learning is used for intelligent agent decision-making through continuous trial and error [19]. Such algorithms include DQN algorithm, Double Deep Q-Network (DDQN) algorithm, etc. In complex dynamic environments, DQN algorithm inherits the advantages of PRM algorithm in processing continuous space, while it also achieves real-time search performance like A* algorithm through neural networks. Due to its excellent performance, many scholars at home and abroad use this complex neural network model to learn unknown environments, and then rely on feedback information to drive model decisions, achieving efficient obstacle avoidance. These studies are of vital significance in improving the performance and adaptability of AGVs.

At present, research regarding the DRL algorithm on path planning algorithms has achieved some breakthroughs, but there are also some problems and shortcomings; for example, the DRL algorithm has too many iterations, a slow convergence speed, and poor practicality. Although these problems have been optimized to some extent, there are still significant limitations, especially in unknown environments [20]. Liu et al. [21] attempted to propose a path planning method based on reinforcement learning, establishing a robot motion accuracy model and transforming it into a Markov Decision Process (MDP) to improve the path accuracy and smoothness. Demelash et al. [22] propose a hybrid algorithm that combines Dueling DQN, priority experience replay, and customized reward functions, providing a new solution for autonomous navigation for robots in complex environments. He et al. [23] effectively improved the global optimization ability of time-varying road network path planning through the design of a DDQN algorithm and dynamic reward. Sun et al. [24] combined the global path planning ability of the artificial potential field method with DDQN to solve the problems of APF being prone to falling into local optimality and there being too much blind exploration in traditional DDQN. Li et al. [25] conducted in-depth research on the Deep Q-Network (DQN) algorithm that integrates Navigation Priority (NP) and Priority Experience Replay (PER) strategies, providing a new solution for the intelligent path planning problem in marine environments. El Wafi et al. [26] propose a method for the adaptive adjustment of hyperparameters to address the path planning problem in complex maze environments, solving the problem of poor adaptability caused by fixed parameters in traditional reinforcement learning. However, further solutions are needed to address issues such as the slow convergence speed, susceptibility to local optima, and poor generalization ability in unknown environments or large-scale scenarios. Therefore, an improved DQN path planning algorithm based on priority experience replay and the Boltzmann strategy, named the B-PER DQN algorithm, is proposed in this paper.

In the second part of this paper, the temperature parameter in the Boltzmann strategy is introduced into the DQN algorithm, and an improved B-PER DQN algorithm model based on preferential empirical replay is established. In the third part, a multi-dimensional reward function is constructed, and the path planning strategy for the B-PER DQN algorithm is designed. In the fourth part, the results for the DQN, DDQN, and B-PER DQN algorithms are compared through simulation experiments, and the performance of B-PER DQN algorithms in terms of the convergence speed and path planning effect is analyzed. In the fifth part, the advantages and disadvantages of the mixed-action selection strategy with temperature parameters and the challenges faced by the algorithm in the real environment are discussed. Finally, in the sixth part, the conclusions are presented and analyzed, finding that the algorithm outperforms the comparative algorithm in static grid environments of different scales, and the future research directions are discussed.

## 2. AGV Path Planning Problem

### 2.1. Description of Path Planning Problem

**Figure 1:**
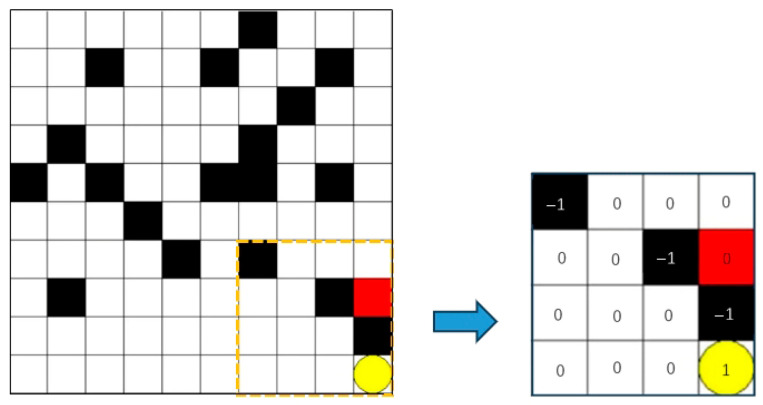
Simulation of the grid environment of an AGV.

The grid environment for the AGV path planning problem is shown in Figure 1. In the figure, black represents the impassable obstacles of the AGV with the state value of −1, white represents the passable passage with the state value of 0, red square represents the AGV, and yellow circle represents the end point with the state value of 1. The AGV can only appear within a grid and move between four adjacent grids.

The goal in the path planning problem is to find a path N consisting of a series of adjacent white cells after being given the starting point $n_{S}$ and the accessible end point $n_{F}$ and to try to keep the path length L(N) as short as possible, as shown in Equation (1):

(1) $$\begin{array}{l} N=\left(n_{S},n_{F}\right)=\left(n_{S},neighbor\left(n_{S}\right),neighbor\left(neighbor\left(n_{S}\right)\right)\ldots n_{F}\right)\\ L\left(N\right)=\left|N\right|-1 \end{array}$$

In Equation (1), $neighbor(n_{S})$ represents the adjacent node of node $n_{S}$. L(N) is the length of path N.

### 2.2. Action Space Design

**Figure 2:**
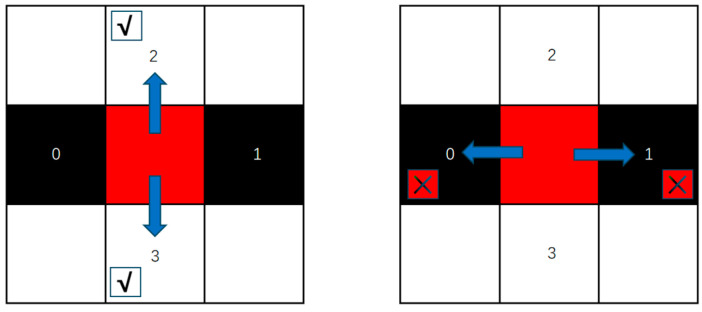
AGV motion direction and coding.

Based on the natural characteristics of the grid environment, the AGV can only move in four directions, as shown in Figure 2, the arrow indicates the direction of advance, the cross symbol indicates that passage is not allowed. Its action space is $\left\{0,1,2,3\right\}$, and the four actions are mapped as integers 0, 1, 2, and 3, respectively, through simple integer encoding. The behavior of the AGV conforms to the rules of the maze and avoids invalid or illegal movements, including the following main points: it cannot move beyond the boundaries of the maze; it cannot move to a cell where an obstacle is located; and the action will stop when the AGV reaches the target location.

### 2.3. State Space Design

The state space is the collection of all possible states of agents in the environment and the core representation of the AGV’s perception of the environment in the path planning process. It defines all relevant information that agents can observe at each time step. On the traditional grid map, the location of the AGV can be determined using the horizontal and vertical coordinates of its grid point. This shows that the state space of the AGV is two-dimensional, and its scope mainly depends on the size of the map. In this case, AGV motion planning is mainly based on its position information on the map.

In the improved algorithm, in order to perceive the surrounding information more comprehensively, the network input is expanded to a six-dimensional AGV state. The first two dimensions are used to represent the relative positional relationship between the current position of AGV and the end point. By describing the azimuth relationship between the current position and the end point, a clear direction of advance is provided for the AGV. The last four dimensions are used to describe the environmental information around the grid point where the AGV is located. These four dimensions, respectively, represent the values of four adjacent grid points (up, down, left, and right) around the AGV, reflecting the characteristics of the surrounding environment, such as whether there are obstacles or other AGVs. They help the AGV to perceive changes in the surrounding environment in real time and effectively avoid obstacles and ensure its safety and navigation efficiency in complex environments. The state vector s is shown in Equation (2):

(2) $$\begin{array}{l} P_{x}=\left\{\begin{array}{c} 0,x_{A}=x_{F}\\ \frac{x_{A}-x_{F}}{\left|x_{A}-x_{F}\right|},otherwise \end{array}\right.\\ P_{y}=\left\{\begin{array}{c} 0,y_{A}=y_{F}\\ \frac{y_{A}-y_{F}}{\left|y_{A}-y_{F}\right|},otherwise \end{array}\right.\\ E_{i}=M\left({n^{\prime }}_{A}\left(a_{i}\right)\right),i\in \left[0,3\right]\\ s=\left[P_{x},P_{y},E_{0},E_{1},E_{2},E_{3}\right] \end{array}$$

In Equation (2), $x_{A}$ and $x_{F}$ are the abscissa values of the current AGV and the end point, respectively; $y_{A}$ and $y_{F}$ are the ordinate values of the current AGV and the end point, respectively; $P_{x}$ and $P_{y}$ are the abscissa and ordinate orientational relationships relative to the target position, respectively; M( ) represents the state function of the environment and outputs the state value of the current position according to the position $E_{i}$; $a_{i}$ is the action value of the AGV action space; ${n^{\prime }}_{A}(a_{i})$ indicates the position of the AGV after taking action $a_{i}$; and s is the state vector.

Improving the input design of the algorithm and incorporating the environmental information around the AGV not only improves the path planning capability of the AGV but also enhances its adaptability and flexibility in complex environments.

### 2.4. Multi-Objective Reward Function Design

The reward function is a core concept in reinforcement learning and plays a vital role in the interaction between the agent and the environment. The value of the reward function determines the value evaluation of different state–action pairs by the agent. In this improved algorithm, the reward function consists of four parts:

1. $r_{1}$ is the comparison value of the Manhattan distance between the current state and the previous state from the endpoint. If the distance decreases, then a reward value is set based on the reduced distance; otherwise, set it to −1.
2. $r_{2}$ is the reward for reaching the endpoint. If it is reached, set it to 50; otherwise, set it to −1. This can provide positive incentives to encourage the agent to reach the endpoint faster and accelerate the convergence of the training process.
3. $r_{3}$ is the obstacle punishment, which includes two situations: encountering obstacles in the environment and moving outside the boundaries of the environment. If the agent collides with an obstacle, set it to −5; otherwise, set it to 0. This can reduce the agent’s ineffective exploration near the obstacle by punishing it for collision with the obstacle. Learning to avoid the obstacle is a better strategy.
4. $r_{4}$ is the step penalty. Each move will be slightly penalized, so set it to −0.1. The continuous accumulation of $r_{4}$ can motivate the agent to minimize the number of steps, prevent it from wandering around the map for a long time, and achieve the goal faster.

The final reward function is derived from the addition of the four parts, as shown in Equation (3):


(3) $$\begin{array}{l} R=\rho (r_{1}+r_{2}+r_{3}+r_{4})\\ r_{1}=\left\{\begin{array}{c} 1,\left(\left|x_{A}-x_{F}\right|+\left|y_{A}-y_{F}\right|\right)<\left(\left|{x^{\prime }}_{A}-x_{F}\right|+\left|{y^{\prime }}_{A}-y_{F}\right|\right)\\ -1,\left(\left|x_{A}-x_{F}\right|+\left|y_{A}-y_{F}\right|\right)\geq \left(\left|{x^{\prime }}_{A}-x_{F}\right|+\left|{y^{\prime }}_{A}-y_{F}\right|\right) \end{array}\right.\\ r_{2}=\left\{\begin{array}{c} 50,n_{A}=n_{F}\\ -1,n_{A}\neq n_{F} \end{array}\right.\\ r_{3}=\left\{\begin{array}{c} -5,collide\\ 0,otherwise \end{array}\right.\\ r_{4}=-0.1\times step \end{array}$$

In Equation (3), a is the action performed by the AGV; A represents the attributes of the AGV at this time; F represents the endpoint; and ${x^{\prime }}_{A}$ and ${y^{\prime }}_{A}$ represent the horizontal and vertical coordinate values of the AGV after movement, respectively. To ensure the stability of the reward function, set parameter ρ=h+d and scale it proportionally in different scale environments, where h and d represent the length and width of the map, respectively.

## 3. Improved DQN Algorithm

### 3.1. Boltzmann Strategy

The Boltzmann strategy is a classic exploration strategy used in reinforcement learning. In reinforcement learning, the agent learns the optimal strategy by interacting with the environment to maximize the cumulative reward. The agent needs to find a balance between exploration and utilization. Exploration refers to attempting new actions to obtain more information about the environment, and utilization refers to selecting the optimal action based on the information currently known.

As an effective balancing strategy, the Boltzmann strategy regulates the degree of exploration and utilization by introducing a temperature parameter [27]. The Boltzmann strategy is based on the Boltzmann Distribution, and its core purpose is to calculate the probability of each action being selected based on its value function [28]. The probability $P\left(a\left|s\right.\right)$ of action a being selected in state s can be expressed as follows:

(4) $$P\left(a\left|s\right.\right)=\frac{\exp\left(Q\left(s,a\right)/\tau \right)}{\sum_{a^{\prime }\in A}\exp\left(Q\left(s,a^{\prime }\right)/\tau \right)}$$

In Equation (4), $Q\left(s,a\right)$ is the estimated value of the action a in the state s; τ is the temperature parameter; $a^{\prime }$ represents the next action, and A represents the action space. When τ is high, the selection probability of all actions tends to be uniform; that is, the strategy is more inclined to random exploration. When τ is lower, the strategy is more inclined to choose the action with higher value; that is, it is closer to a greedy strategy.

With the development of deep learning, the Boltzmann strategy has also been further improved and optimized in DRL. In order to encourage the agent to perform more diverse actions, entropy regularization terms are added to the loss function to improve the exploration efficiency. During training, the temperature parameter τ can be dynamically adjusted according to the agent’s learning progress. By setting the temperature parameter reasonably and adding entropy regularization, the learning efficiency of the agent can be improved.

### 3.2. Priority Experience Replay

In reinforcement learning, experience replay is a key technology that breaks the correlation of data and improves learning efficiency by storing and replaying experiences. However, traditional experience replay uses uniform sampling, ignoring the importance of different experiences. Priority experience replay solves this problem by introducing a priority sampling mechanism so that the agent can learn more efficiently [29]. The core purpose of priority experience replay is to sample the importance of experience (usually measured by Temporal Difference (TD) error). The priority of each experience is determined based on its TD error. The larger the TD error, the higher the importance of the experience. The sampling probability $p_{i}$ is defined as follows:

(5) $$\begin{array}{l} P\left(i\right)=\frac{p_{i}^{\alpha }}{\sum_{k}p_{k}^{\alpha }}\\ w_{i}={\left(\frac{1}{C}\cdot \frac{1}{P\left(i\right)}\right)}^{\beta } \end{array}$$

In Equation (5), $p_{i}$ is the priority of the i-th experience; C represents the historical path data stored in the experience replay buffer; α is used to control the degree of influence of the priority; $w_{i}$ is the importance sampling weight, which is used to reduce the sampling bias; and β is used to adjust the impact of the weight.

Priority experience replay is an effective reinforcement learning technique, which significantly improves the learning efficiency and convergence speed by prioritizing the sampling of important experiences. In recent years, priority experience replay technology has been continuously developing. Researchers have proposed several methods of improvement, such as proportion-based prioritization and sort-based prioritization. It performs well in various applications, and its application scope will be further expanded on.

### 3.3. Improved DQN Algorithm Model

The DQN algorithm is an important algorithm in the field of reinforcement learning; it combines deep learning and Q-learning so that Q-learning can be effectively applied in high-dimensional spaces [30]. It also introduces experience replay and a two-layer network structure to improve the stability and efficiency of training [31]. The two-layer network structure of the DQN includes a current Q-network and a target network. The parameters of the target network are updated in a certain time interval to reduce the fluctuation in the training process, to improve the stability of the training.

In the DQN algorithm, the impact of an agent’s actions in a specific state on the subsequent process is measured using a Q-value, and the value of the current action is evaluated based on the immediate reward obtained and the potential value of the subsequent state. The Q-value is recorded in the Q-table and will be continuously updated as the agent interacts with the environment. When the Q-table is iteratively updated, the agent determines the actions that it will perform based on the highest Q-value at each moment. The Q-function Q(s,a) represents the expected cumulative reward that can be obtained after taking action a in state s, and the equation is as follows:

(6) $$Q_{\pi }\left(s,a\right)=E_{\pi }\left[G_{t}\left|S_{t}=s,A_{t}=a\right.\right]$$

(7) $$Q\left(s,a\right)\leftarrow Q\left(s,a\right)+\alpha \left(r+\gamma \underset{a^{\prime }}{\max Q\left(s^{\prime },a^{\prime }\right)-}Q\left(s,a\right)\right)$$

In Equations (6) and (7), E represents the expected value; G is the return function, which is a measure of the subsequent impact of action a in state s; Q(s,a) is the Q-value of the current state-action pair; α is the learning rate; r is the immediate reward; γ is the discount factor; and $\max Q(s^{\prime },a^{\prime })$ is the maximum Q-value in the next state.

The strategy of the DQN is completely determined by the Q-value. It has poor adaptability in complex environments, and there is a natural imbalance between exploration and utilization. To solve this problem, a B-PER DQN algorithm is constructed in this paper, which adopts a hybrid action selection strategy combining ε-greedy strategy and Boltzmann strategy exploration mechanisms.

Firstly, the temperature parameter τ is introduced to dynamically adjust the randomness of exploration, thereby improving the learning efficiency and adaptability of the agent. The action selection probability of the hybrid strategy is shown in Equation (8):

(8) $$P\left(a\left|\left.s\right)\right.\right.=\left\{\begin{array}{c} \frac{\varepsilon }{\left|A\right|},\partial <\varepsilon \\ \frac{\exp\left(Q\left(s,a\right)/\tau \right)}{\sum_{a^{\prime }\in A}\exp\left(Q\left(s,a^{\prime }\right)/\tau \right)},otherwise \end{array}\right.$$

In Equation (8), $Q(s,a^{\prime })$ represents the expected return of action $a^{\prime }$ in state s, and ∂ is a random number of (0, 1). The parameters ε and τ are constantly changing, as shown in Equations (9) and (10).

(9) $$\varepsilon =\max\left(\varepsilon \times \varepsilon_{delay},\varepsilon_{\min}\right)$$

(10) $$\begin{array}{c} \tau_{t+1}=\left\{\begin{array}{c} \begin{array}{cc} \max\left(0.95\times \tau_{t}<0.1\right) & if\;\forall r\in recent\_r_{t},r\geq avg\_r_{t} \end{array}\\ \begin{array}{cc} \max\left(1.05\times \tau_{t}<0.1\right) & if\;\forall r\in recent\_r_{t},r\leq avg\_r_{t} \end{array}\\ \begin{array}{cc} \tau_{t} & otherwise \end{array} \end{array}\right.\\ avg\_r_{t}=\frac{1}{W}\sum_{k=t-W+1}^{t}r_{k}\\ recent\_r_{t}=\left\{\begin{array}{c} \begin{array}{cc} recent\_r_{t-1}\cup \left\{\left.r_{t}\right\}\right. & if\left|recent\_r_{t-1}\right|<W \end{array}\\ \begin{array}{cc} \left(recent\_r_{t-1}\backslash \left\{\left.r_{t-W}\right\}\right.\right)\cup \left\{\left.r_{t}\right\}\right. & otherwise \end{array} \end{array}\right. \end{array}$$

In Equation (9), $\varepsilon_{delay}=0.995$,$\varepsilon_{\min}=0.2$. At the beginning, ε=1, decay it exponentially with $\varepsilon_{delay}=0.995$. If the number of rounds is less than 100, decay it to the lowest $\varepsilon_{\min}=0.2$ to prevent premature stopping of exploration; otherwise, it will continue to decline.

In Equation (10), t is the training round; $r_{t}$ is the cumulative reward of the t round; $avg\_r_{t}$ is the average reward for the last W round; $recent\_r_{t}$ is the reward for the last W rounds at the end of the t round; and W is the size of the reward window set to 10. When t<10, the reward is accumulated directly; when t≥10, $r_{t-10}$ is replaced by $r_{t}$.

Secondly, the PER mechanism is introduced to measure the priority of sampling data with the TD error as the standard. The calculation method is shown in Formula (5) in Section 3.2.

Meanwhile, in the B-PER DQN algorithm, the core loss function of the DQN algorithm is retained, which minimizes the deviation between the target value and the output value of the neural network [32]. The loss function is defined as follows:

(11) $$L\left(\theta \right)=E_{\left(s_{t},a_{t},r_{t},s_{t+1}\right)}∼D\left[{\left(y_{t}-Q\left(s_{t},a_{t};\theta \right)\right)}^{2}\right]$$

In Equation (11), L(θ) is a loss function that measures the difference between the Q-value of the Q-network output and the target Q-value; D is a sample in the experience replay buffer; $Q(s_{t},a_{t};\theta )$ is the output of the current Q-network; and $y_{t}$ is the target Q-value, with its calculation method shown in Equation (12).

(12) $$y_{t}=r_{t}+\gamma \underset{a^{\prime }}{\max Q\left(s_{t+1},a^{\prime };\theta^{-}\right)}$$

In Equation (12), $r_{t}$ is an instant reward after taking action $a_{t}$ in state $s_{t}$, and $Q(s_{t+1},a^{\prime };\theta^{-})$ is the maximum Q-value of all possible actions from the next state $s_{t+1}$, calculated using the parameters $\theta^{-}$ of the target network.

In addition, the motion direction and state dimensions of the AGV are customized based on the simulated environment of AGV operation set in Section 2.1, including obstacles and paths, to evaluate the expected rewards for taking specific actions in the current state. Specific representations of the action selection strategy and state vector are shown in Equations (2) and (8).

**Figure 3:**
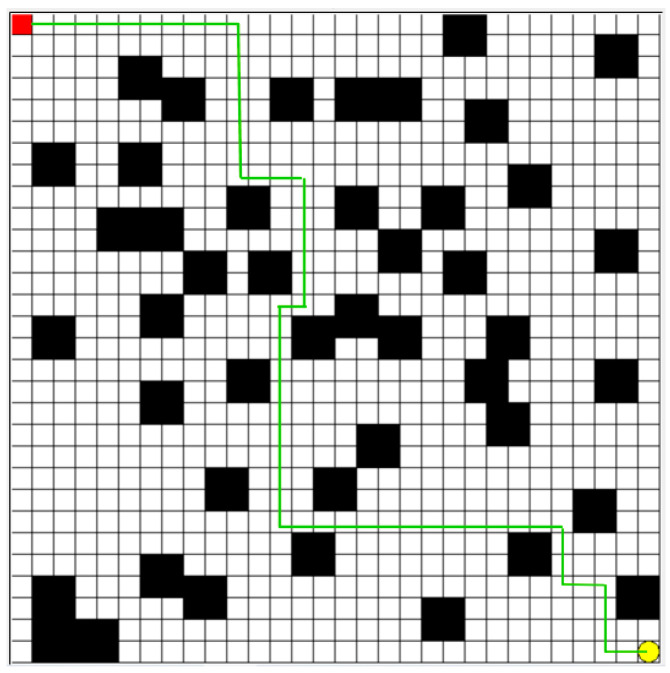
Preset path planning for AGV departure.

Finally, the trained model is integrated into the AGV control system to obtain the preset departure path, as shown by the green line in Figure 3. During the training phase, the real-time operation status and path planning effectiveness of the AGV are monitored, and feedback data are continuously collected. Based on this real-time feedback information, the strategy parameters are adjusted, or the model is retrained to continuously improve the performance and adaptability of the AGV.

### 3.4. The Overall Framework of the B-PER Algorithm

**Figure 4:**
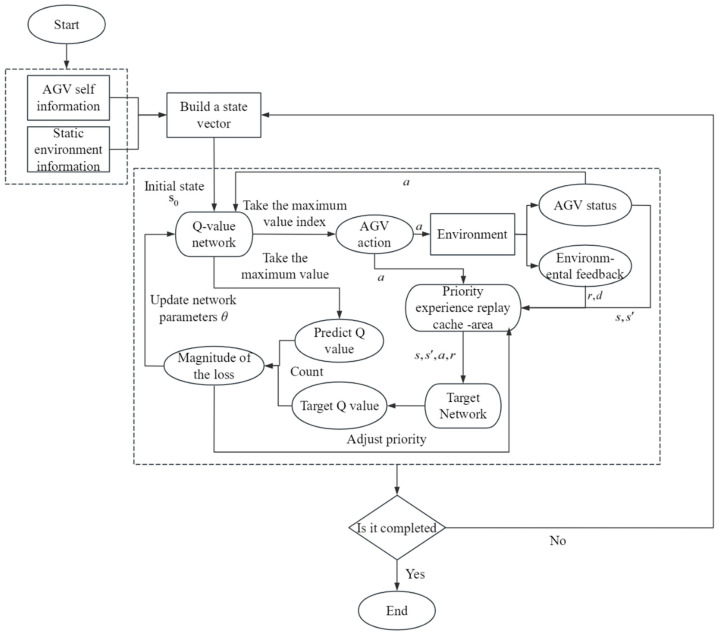
Algorithm flow framework.

The overall flow of the B-PER DQN algorithm proposed in this paper is shown in Figure 4. The block in the figure represents the component in the algorithm, and the ellipse represents the specific parameter values input to or output from the component. Firstly, after the environmental data is collected and modeled, the AGV obtains its own state and environmental information in real time through the sensor to an accurate state vector. Subsequently, the Q-value network is trained using an experience replay mechanism and target network to improve the strategy parameter θ in the AGV running continuously in the environment as well as the accuracy and efficiency of its path planning. In addition, the traditional ε-greedy strategy and Boltzmann strategy are combined to dynamically adjust the exploration intensity through temperature parameters. At the same time, the movement, obstacle avoidance, path length, distance change, and destination reward are integrated, and the sampling priority is dynamically adjusted according to the TD error to improve the utilization rate of key samples and guide the AGV to reach the target position safely and efficiently.

The specific algorithm is shown in Algorithm 1.
**Algorithm 1:** B-PER DQN algorithm1. Initialize the priority experience playback buffer D, the capacity is C
2. Initialize environment parameters and training parameters
3. For episode =1 to M do
4. Reset the environment, get the initial states, initialize episode reward =0
5.  While not done:
6.   Select the action according to Equation (8).
7.   Execute the action a and store $s,s^{\prime },r,d$ to D
8.   If C > 256:
9.   According to Equation (5), the sample of batch-size size is extracted from D
10.   Calculate and update network parameters according to Equation (11)
11.   End if
12.  Dynamically adjust τ according to Equation (10)
13.  Attenuation ε according to Equation (9)
14. End for

## 4. Simulation Experiments

### 4.1. Design of Experiments

**Figure 5:**
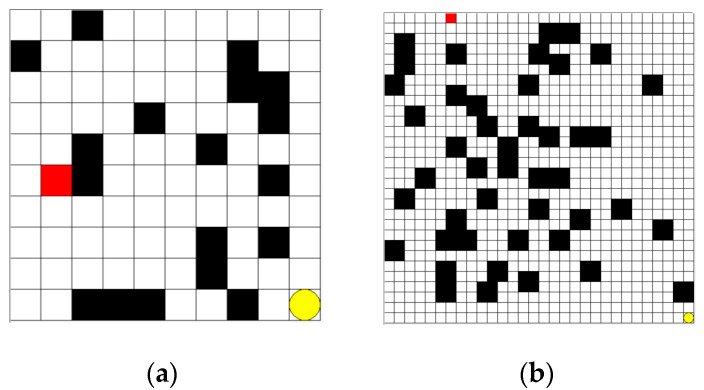
Environment maps of different scales: (**a**) 10 × 10; (**b**) 30 × 30.

In order to comprehensively evaluate the level of excellence of the performance of the improved DQN algorithm in convergence speed and path planning, two sets of experiments were designed. Firstly, the logistics warehouse environment was divided into a square grid of the same size to represent the warehouse information map, and the size was set as 10 × 10 for a simple environment map and 30 × 30 for a complex environment map, as shown in Figure 5. The B-PER DQN algorithm, the traditional DQN algorithm, and the DDQN algorithm were trained in 500 rounds, respectively, and the results were analyzed and compared. Through the detailed analysis of the data during the training process, the significant advantages of the B-PER DQN algorithm in terms of convergence speed were highlighted. Secondly, in order to verify the performance of the algorithm in path planning, the B-PER DQN algorithm, the traditional DQN algorithm, and the DDQN algorithm were also trained for 500 rounds on the 10 × 10 and 30 × 30 maps. Key performance indicators, such as the path length and completion rate, were compared to comprehensively evaluate the actual performance of the new method. All experiments in this section were simulated in Python 3.7 based on Anaconda 3.7.

### 4.2. Comparative Experiment and Analysis of Convergence Speed

**Figure 6:**
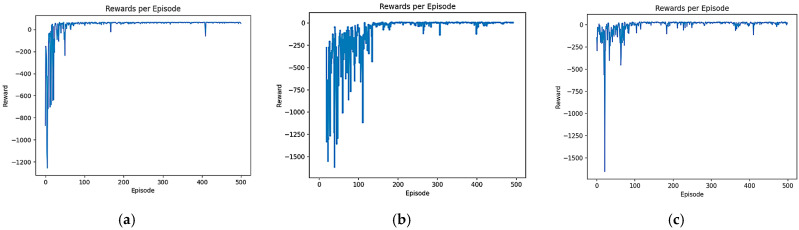
Comparison of the reward function of B-PER DQN, DQN, and DDQN in the 10 × 10 environment: (**a**) B-PER DQN; (**b**) DQN; (**c**) DDQN.

**Figure 7:**
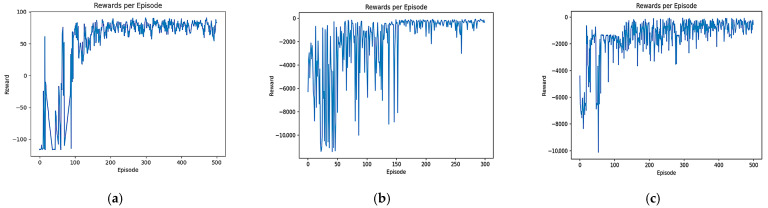
Comparison of the reward function of B-PER DQN, DQN, and DDQN in the 30 × 30 environment: (**a**) B-PER DQN; (**b**) DQN; (**c**) DDQN.

In order to test the convergence speed advantage of the B-PER DQN algorithm, a comparative experiment was set up and the training input parameters were set according to Table 1. A total of 500 rounds of training were performed for the B-PER DQN, DQN, and DDQN algorithms in the 10 × 10 and 30 × 30 grid map environments with an obstacle area of about 20%, as shown in Figure 5. The learning rate determines the amplitude of the model update, which has an important impact on the convergence speed and stability. Appropriately reducing the learning rate according to Table 1 can accelerate the convergence speed and improve the training efficiency. The number of training sessions determines the adequacy of model training, ensuring that the model has enough time to learn and adapt to the environment. In addition, exploration strategy parameters, such as temperature parameters, can affect the exploration behavior of the agent in the environment. The dynamic adjustment of temperature parameters in the environment can balance exploration and utilization, help agents discover new paths and strategies, and prevent a fall into local optimization. The reward function determines the influence of different reward items on the agent’s behavior. The reasonable setting of the movement reward, distance reward, collision penalty, endpoint reward, and step number penalty can encourage exploration in the early stage of training. In the early stages, random exploration by intelligent agent may lead to instability in reward signals and agent behavior, resulting in drastic fluctuations in reward function changes. However, as the agent’s understanding of the environment increases, its desire to explore gradually decreases and tends towards stability, thereby achieving efficiency and safety in obstacle avoidance and path planning. The training results are shown in Figure 6 and Figure 7. Figure 6 shows a comparison of the reward function changes in the three algorithms in each round in the 10 × 10 environment. Figure 7 shows the comparison of the reward function changes in the three algorithms in each round in the 30 × 30 environment.

As shown in Figure 6, although the reward value of each algorithm is negative in a 10 × 10 simple environment at the beginning of training, the convergence speed of all three algorithms is relatively fast. After the introduction of the improved temperature parameter, the B-PER DQN algorithm began to gradually stabilize after 50 rounds of training and fully converged after 100 rounds, while the other two algorithms could not fully converge until after 150 rounds. Compared with the simple and regular 10 × 10 environment, the convergence time of the model took longer in the more complex environment. As shown in Figure 7, in terms of cumulative reward values of each round, the traditional DQN algorithm gradually stabilizes after around 150 rounds, while the B-PER DQN algorithm begins to converge rapidly around about 100 rounds of training, and the convergence speed of the B-PER DQN algorithm has increased by nearly 33.3% compared to the traditional DQN algorithm. The DDQN algorithm also achieved good convergence around 100 rounds, but it was far less stable than the B-PER DQN algorithm before 150 rounds of training. The convergence of the cumulative reward values of B-PER DQN and DDQN was better than that in the traditional DQN algorithm, which shows that their optimization of the reward function is fruitful.

According to the above figures, the B-PER DQN algorithm effectively addresses the problems of slow convergence speed, low efficiency, and poor stability encountered in traditional DQN algorithms and significantly improves the convergence speed.

### 4.3. Comparative Experiment and Analysis of Path Planning Effect

In the following experiment, the AGV was controlled to perform tasks 500 times using the B-PER DQN algorithm, DQN algorithm, and DDQN algorithm after they had completed 500 rounds of training in the map environment shown in Figure 4. Then, experimental analysis was conducted on the training steps and successful execution times of the working paths of the three algorithms in the above tasks. The completion rate directly reflects the algorithm’s ability to achieve task objectives. A high completion rate indicates that the agent has mastered an effective strategy, while a low completion rate exposes policy flaws or environmental complexity. SR denotes the proportion of agents that successfully achieve the task objective during reinforcement learning training, as shown in Equation (13).

(13) $$\begin{array}{l} SR=\frac{\sum_{\chi =0}^{k}f_{\chi }}{k+1},\left(0\leq k\leq episodes\right)\\ f_{\chi }=\left\{\begin{array}{c} 1,if\varphi_{\theta }\left(\cdots \varphi_{\theta }\left(\varphi_{\theta }\left(n_{G}\right)\right)\right)=n_{F}\\ 0,otherwise \end{array}\right. \end{array}$$

In Equation (13), k is the number of rounds completed. $\varphi_{\theta }$ is the model strategy, and its value of $f_{x}$ is set to 1 if the model strategy can reach the end point from the initial point within a given number of steps; otherwise, it is set to 0.

#### 4.3.1. Comparison Experiment for Path Length

In this experiment, the performance of the DQN, DDQN, and B-PER DQN algorithms in AGV path planning was compared among 500 tasks, and the path length and stability of each task were evaluated. In our evaluation of the performance of the AGV path planning algorithm, path length was the core indicator for measuring the task execution efficiency. The shortening of the path length is directly related to the AGV’s handling speed improvement and reduction in energy consumption, which are crucial in the optimization of operation efficiency in scenarios such as logistics and warehousing. At the same time, the stability of the algorithm can be quantified based on the standard deviation of the path length, which reflects the planning consistency of the algorithm in different task environments. An algorithm with high stability can better adapt to scene changes and reduce path fluctuations caused by environmental disturbances to ensure reliability in planning results. Through a comprehensive analysis of the path length and stability of the three algorithms, the actual performance of AGV path planning can be systematically evaluated. This evaluation framework not only provides a basis for algorithm selection in specific scenarios but also guides the way for the subsequent iterative optimization of algorithms, such as enhancing stability for complex dynamic scenarios or preferentially compressing the path length in a fixed process.

**Figure 8:**
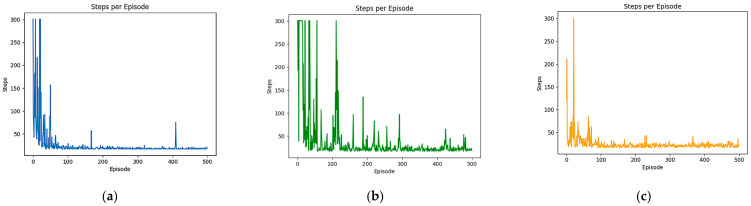
Comparison of the path lengths of B-PER DQN, DQN, and DDQN in the 10 × 10 environment: (**a**) B-PER DQN; (**b**) DQN; (**c**) DDQN.

As shown in Figure 8 and Table 2, the fluctuation range of the length of each task path of the DQN algorithm is between 18 and 300, the average value is stable at 34.7, and the standard deviation is 42.77 in the simple environment, indicating that the algorithm has certain fluctuations during the training process. In contrast, the fluctuation range of the path length of the DDQN algorithm decoupling the action selection from the Q-value evaluation has no significant change. However, the mean is reduced to 27.52 and the standard deviation is reduced to 29.99, and stability is strengthened. This may be because DDQN only uses the target network when evaluating the Q-value, while the action selection is performed through the online network. The B-PER DQN algorithm, which uses the temperature parameter improvement exploration strategy, has achieved remarkable results in optimizing the path planning effect. Its average value is reduced to 24.35, which is 29.83% shorter than the DQN algorithm and 11.52% shorter than the DDQN algorithm. At the same time, the standard deviation of the algorithm is only 24.80, which is the lowest among the three algorithms, indicating that this algorithm has the strongest stability.

**Figure 9:**
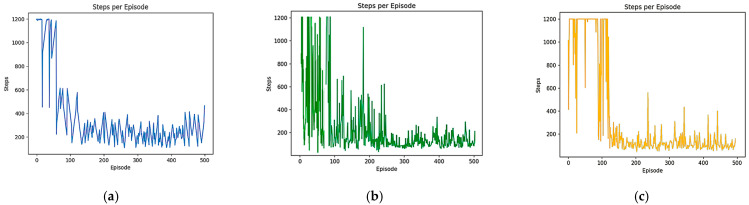
Comparison of the path lengths of B-PER DQN, DQN, and DDQN in the 30 × 30 environment: (**a**) B-PER DQN; (**b**) DQN; (**c**) DDQN.

As shown in Figure 9 and Table 3, the median length of each task path in the DQN algorithm is 339 grid edge lengths, the average value is stable at 484.66, and the standard deviation is 378.01 in the complex environment. The algorithm has certain fluctuations during the training process. In contrast, the median path length in the DDQN algorithm is reduced to 278 grid edge lengths, and the mean and standard deviation are also reduced to varying degrees. The improved B-PER DQN algorithm has the best effect in optimizing the path planning effect. Its median has decreased to 260, and its standard deviation is also the lowest, reduced by 36.64% compared to the DQN algorithm and 19.81% compared to the DDQN algorithm. With the increasing scale and complexity of the environment, the advantages of the B-PER DQN algorithm in path planning are becoming more and more significant.

In summary, the B-PER DQN algorithm has shown superior performance and stability in path planning, providing strong support for future research work.

#### 4.3.2. Comparative Experiment of Completion Rate

In the path planning task, the completion rate is a key indicator in measuring the number of times that the agent successfully reaches the target, which intuitively reflects whether the path planning algorithm can find a feasible path in a complex environment. Therefore, the completion rate is important in improving the overall performance. First of all, path planning needs to strike a balance between exploring unknown areas and known paths. A high early exploration rate can help in discovering potential paths, while a high utilization rate later on can consolidate successful experiences. The completion rate increases steadily, indicating that the algorithm achieves a dynamic balance and avoids the problems of premature convergence and blind exploration. In addition, the completion rate can be used to effectively evaluate the robustness of the algorithm in an environment with dense obstacles or dynamic changes. Moreover, the trend in the completion rate can provide a basis for hyperparameter tuning. By combining the completion rate with the path length and cumulative reward, the multi-dimensional evaluation can more comprehensively reflect the comprehensive performance of path planning.

**Figure 10:**
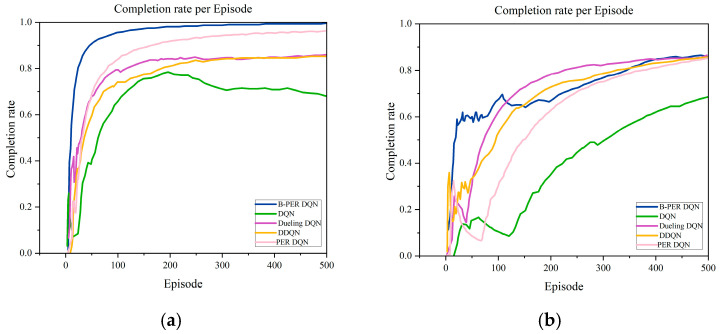
Comparison of the completion rate of B-PER DQN, DQN, DDQN, Dueling DQN, and PER DQN: (**a**) 10 × 10; (**b**) 30 × 30.

The experiments in this section were based on a randomly generated 10 × 10, 30 × 30 grid environment, as shown in Figure 4. In order to verify the performance of the improved algorithm, the B-PER DQN algorithm, conventional DQN algorithm, DDQN algorithm, Dueling DQN algorithm, and PER DQN algorithm are compared in this section, and the training results are shown in Figure 10. Figure 10 shows a comparison of the completion rate of each round of the five algorithms in the 10 × 10 environment and the 30 × 30 environment, respectively.

As shown in Figure 10, the B-PER DQN algorithm proposed in this paper shows an excellent initial performance in the 10 × 10 environment and tends to stabilize after about 70 rounds, achieving a significant improvement in completion rate compared to the conventional DQN algorithm. Taking 300 cycles as observation nodes, it improved by 12.5% compared to the DDQN algorithm, 8.4% compared to the PER DQN algorithm, and 5.9% compared to the Dueling DQN algorithm. The specific data are shown in Table 4.

As the environment becomes more complex, in the 30 × 30 environment, as shown in Figure 10, the completion rate of the B-PER DQN algorithm rapidly increases to about 0.65 within 0–100 cycles, significantly higher than the other four algorithms. After 200 cycles, the growth rate slows down, and the completion rate is about 0.86 at 500 cycles. The DQN algorithm initially rises slowly, and the completion rate of the first 100 rounds is only 0.2, which is significantly behind that of the B-PER DQN algorithm. The growth rate accelerates after about 150 rounds, and the final completion rate is about 0.65. However, it is still lower than that of the other four algorithms. The PER DQN algorithm accelerates after 100 cycles, with a final completion rate of approximately 0.8, but the completion rate in the early stage is significantly lower than that of the B-PER DQN algorithm. The completion rates of the DDQN and Dueling DQN algorithms before 100 cycles are lower than that of the B-PER DQN algorithm, indicating that the improved algorithm can learn effective strategies faster and handle various starting conditions and environmental changes more stably. At the mid-term stage, the completion rate of the DDQN and Dueling DQN algorithms is higher than that of the B-PER DQN algorithm. The reason for this is that their exploration strategy in the mid-term of training is not effective enough, which leads to an inability to fully explore all possible state and action combinations in the environment, resulting in a decrease in generalization ability.

In summary, Figure 10 shows the proportion of agents successfully reaching the target after each training of the three algorithms. It can be clearly seen that the blue curve is in the highest position most of the time, which means that the improved B-PER DQN algorithm presented in this paper has obvious advantages in terms of the proportion of task completion. It has demonstrated superior performance in path planning, providing strong support for future research work. However, in the future, the stability issues faced by algorithms in complex environments should be further explored and solutions sought to improve their performance. It should be noted that the current experiments are conducted in an ideal simulation environment, without considering factors such as sensor noise, communication delay, and hardware computing limitations in the real world, which may affect the performance of the algorithm.

## 5. Discussion

In this paper, an improved B-PER DQN algorithm is proposed that introduces temperature parameters to adjust the exploration strategy of traditional DQN algorithms and achieve certain results. By controlling the probability distribution of action selection with the temperature parameter, the agent can explore various actions more evenly in the early stages of training, avoiding premature convergence to the local optima. As training progresses, the temperature parameter gradually decreases, and the probability distribution of action selection gradually concentrates on actions with high Q-values, achieving a smooth transition from exploration to utilization. This not only improves the overestimation problem encountered with traditional DQN algorithms, but also dynamically adjusts the intensity of exploration according to the complexity of the environment and the learning progress of the agent. While DDQN addresses the overestimation problem by separating the networks for action selection and action evaluation, it still adopts the ε-greedy method for its exploration strategy. In complex environments, DDQN may suffer from poor learning performance due to the inaccurate evaluation of action values. The improved B-PER DQN algorithm in this paper selects actions by considering the Q-values of all actions, enabling better adaptation to uncertainties in complex environments.

However, the experimental data in this study primarily originate from simulated environments, and the algorithm’s performance in real, complex environments still requires further verification. Firstly, the laser radar and visual sensors of AGV often exhibit higher noise levels in real-world environments, and SLAM feature mismatches can cause localization and map distortion, resulting in path deviation or collisions. We can use multi-sensor fusion and anti-noise filtering to improve state estimation, and add noise during training to enhance strategy robustness. Secondly, in reality, dynamic obstacles such as pedestrians and other AGVs can lead to delayed avoidance and insufficient flexibility. In the future, we can integrate an online dynamic obstacle prediction module, combined with the real-time operating status and the external working environment of AGV, to achieve real-time path adjustment and obstacle avoidance to solve technical problems such as real-time and accuracy.

## 6. Conclusions

In this study, an improved path planning method based on DQN is proposed to solve the problems of slow convergence and poor adaptability in complex environments. The new B-PER DQN algorithm introduces a dynamic temperature parameter adjustment mechanism, multidimensional state representation, and multi-objective reward functions. The learning efficiency and path planning robustness of the intelligent agent in complex maze environments are significantly improved. Through a simulation analysis of the convergence speed, path length, and successful execution times, the following conclusions can be drawn:

1. In the simple environment, the B-PER DQN algorithm gradually becomes stable after 50 rounds, while the traditional DQN and DDQN algorithms begin to converge after 100 rounds. The path length is reduced by 29.83% compared with the DQN algorithm and 11.52% compared with the DDQN algorithm. The completion rate is increased by 12.5% compared with the DDQN algorithm, 8.4% compared with the PER DQN algorithm, and 5.9% compared with the Dueling DQN algorithm. This shows that the B-PER DQN algorithm converges faster and has higher stability in the cumulative reward function and training steps and verifies the effectiveness of the adaptive temperature parameters and priority experience replay mechanism.
2. In the complex environment, the cumulative reward values of the B-PER DQN algorithm and the DDQN algorithm start to converge after around 100 rounds of training, but the stability of the DDQN algorithm is poor. In terms of path planning effect, the standard deviation of the B-PER DQN algorithm is also the lowest; it is reduced by 36.64% compared with the DQN algorithm and 19.81% compared with the DDQN algorithm. At the same time, the proportion of successful arrival times is also the highest, indicating that the algorithm is more stable.
3. When the environment scale expands and the complexity increases, the completion rate of B-PER DQN decreases by 9.47%, the completion rate of conventional DQN decreases by 12.50%, and the completion rate of DDQN decreases by 10.0%, which indicates that B-PER DQN is more adaptable to changes in environmental complexity.

Through an in-depth analysis of the limitations of existing deep reinforcement learning methods, in this paper, a new algorithm framework was designed to enhance the efficiency and stability of sample learning. We verified that the algorithm is superior to comparative algorithms in static grid environments of different scales. However, there are still aspects that need to be improved in future research. The current experimental setup is a static, single AGV grid environment, which is relatively ideal compared to the real environment. In the future, robust training methods against sensor noise can be developed, and dynamic obstacle prediction modules can be designed to further improve the performance of the algorithm. Meanwhile, future research will focus on experimental design and validation for dynamic obstacles and multi-AGV coordinated operations.

## Figures and Tables

**Table 1:** Training parameters of B-PER DQN.

Parameter	Value	
Discount factor (γ)	0.99	
Learning rate (α)	0.001, using Adam optimizer	
Number of network nodes	Layer 1	(State dimension, 128)
	Layer 2	(128, 128)
	Layer 3	(128, 128)
	Layer 4	(128, Action dimension)
Maximum search steps per epoch	300	
Batch size	32	
Update rate of the objective function parameter	100	
Replay buffer storage size	2000	
Priority weight (α)	0.6	
Importance sampling weight (β)	0.4	

**Table 2:** Parameters related to the path lengths of B-PER DQN, DQN, and DDQN in the 10 × 10 environment.

Parameter	DQN Algorithm	DDQN Algorithm	B-PER DQN Algorithm
Median	21	21	21
Average value	34.70	27.52	24.35
Maximum	300	300	300
Minimum	18	18	18
Standard deviation	42.77	29.99	24.80

**Table 3:** Parameters related to the path lengths of B-PER DQN and conventional DQN and DDQN in the 30 × 30 environment.

Parameter	DQN Algorithm	DDQN Algorithm	B-PER DQN Algorithm
Median	339	278	260
Average value	484.66	401.34	361.00
Maximum	1200	1200	1200
Minimum	68	70	65
Standard deviation	378.01	298.70	219.13

**Table 4:** Data for 500 rounds of key nodes in the 10 × 10 environment when B-PER DQN, DQN, DDQN, Dueling DQN, and PER DQN are employed.

Algorithm	50 Cycle	300 Cycle	500 Cycle
B-PER DQN	0.8	0.9	0.95
DQN	0.4	0.8	0.7
DDQN	0.6	0.8	0.78
Dueling DQN	0.6	0.85	0.85
PER DQN	0.6	0.83	0.86

## Data Availability

The original contributions presented in this study are included in the article. Further inquiries can be directed to the corresponding author.

## Abbreviations

The following abbreviations are used in this manuscript:

AGV	Automated Guided Vehicle
DQN	Deep Q Network
PRM	Probabilistic Roadmap Method
GWO	Grey Wolf Optimizer
WOA	Whale Optimization Algorithm
DRL	Deep Reinforcement Learning
DDQN	Double Deep Q-Network
PER	Priority Experience Replay
MDP	Markov Decision Process
NP	Navigation Priority
B-PER DQN	Boltzmann Strategy-Priority Experience Replay Deep Q Network
TD	Temporal Difference Error
APF	Artificial Potential Field
RRT	Rapidly Exploring Random Trees
